# The influence of age and the presence of prostate cancer on prostate volume, PSA and PSA density

**DOI:** 10.1111/bju.70169

**Published:** 2026-02-23

**Authors:** Jill Rusbridge, Iztok Caglic, Liness Thavaraja, Nikita Sushentsev, Tristan Barrett

**Affiliations:** ^1^ School of Medicine Addenbrooke's Hospital Cambridge UK; ^2^ Department of Radiology Addenbrooke's Hospital and University of Cambridge Cambridge UK

**Keywords:** PSA, PSA density, prostate cancer, prostate, prostate volume, MRI prostate

## Abstract

**Objective:**

To assess prostate volume (PV) changes with age in symptomatic and asymptomatic men with and without clinically significant prostate cancer (csPCa). In symptomatic patients, we additionally analysed the effect of age and csPCa on PSA and PSA‐density (PSA‐D) and compared these to current National Institute for Health and Care Excellence (NICE) recommended PSA age‐range thresholds.

Patients and Methods

This single‐centre retrospective cross‐sectional study included 2512 men: 760 asymptomatic, disease‐free men and 1752 patients referred on a PCa diagnostic pathway. Magnetic resonance imaging‐derived whole‐gland PV was recorded for all patients. A machine‐learning pipeline with k‐fold cross validation modelled relationships between PV and age.

**Results:**

In asymptomatic men (median PV 25.4 mL), the mean PV per age‐group increased non‐linearly with age, from 18.7 mL at an increase of 0.10 mL/year aged 18 years, to 41.3 mL at 0.68 mL/year aged 89 years, with increased rate of change from the age of 48.9 years. Significant positive relationships were shown between PSA and age in patients with and without csPCa (*r* ^2^ = 0.09 vs 0.13, respectively), with PSA increasing by mean 0.17 ng/mL/year across groups. Patients with csPCa had consistently higher PSA levels. PSA‐D showed significant age‐related linear increases in patients with csPCa but remained consistently lower in those without csPCa at all ages (0.10–0.11 ng/mL^2^), allowing differentiation at a threshold of >0.15 ng/mL^2^.

**Conclusion:**

In asymptomatic men, PV changed non‐linearly with age. Age‐related PSA thresholds are supported; however, a static PSA‐D threshold of 0.15 ng/mL^2^ can be applied across all age ranges.

**Abbreviations**
NICE: National Institute for Health and Care Excellence
(cs)PCa: (clinically significant) prostate cancer
PSA‐D: PSA density
PV: prostate volume

## Introduction

Prostate cancer (PCa) is the most common male cancer in the UK and is the fifth highest cause of mortality for men worldwide [1]. Furthermore, both PCa incidence and mortality have been shown to increase with age [2], with incidence expected to increase with an ageing global population.

The first investigation performed when PCa is suspected clinically, is a PSA test. PSA alone has a low specificity for the diagnosis of PCa and may be raised due to BPH [3], increased age, infection, inflammation, and trauma [4]. The increase of PSA with age is thought to be driven by an increase in prostate size in the ageing male, and particularly an increase in the quantity of PSA‐producing glandular tissue, alongside a breakdown of the physiological barriers restricting leakage of PSA from within the prostatic ductal system [5]. This has led to the adoption of age‐specific PSA thresholds as recommended by the National Institute for Health and Care Excellence (NICE) [6]; however, these have not been accurately mapped in the existing literature [7, 8].

In the current PCa diagnostic pathway, a PSA test is followed by a prostate MRI [6] to determine the need for subsequent biopsy and enable accurate gland volume assessment. With prostate volume (PV), PSA density (PSA‐D) can be calculated, which has been shown to be more accurate than PSA alone [9, 10], and can be used to augment clinical decision making. However, for PSA‐D, age‐related thresholds have not yet been established. Furthermore, it is unclear how PV differs between men that are asymptomatic and disease‐free, symptomatic and disease‐free, or patients with indolent or clinically significant PCa (csPCa). Addressing this knowledge gap may lead to a more personalised use of PSA or even PSA‐D thresholds specific to age, rather than a universal threshold.

In this study, we assessed age‐specific changes in MRI‐derived PV in a large cohort of symptomatic and asymptomatic men with and without PCa to derive age‐specific PSA and PSA‐D threshold levels.

## Patients and Methods

This single‐centre retrospective study was approved by the local Ethics Committee (anonymised), with the need for written informed consent waived. Patient populations were defined as below (Fig. S1). The symptomatic cohort included patients referred to urology with a suspicion of PCa based on LUTS, and/or raised PSA, and/or an abnormal DRE. The asymptomatic cohort had no known prostatic symptoms or LUTS, and no previous investigations or treatment for benign of malignant prostatic disease.

### Asymptomatic, Disease‐Free Men (Cohort 1)

We performed a search of the local electronic medical record system for male patients aged 18–89 years undergoing MRI of the pelvis for any indication between January 2019 to December 2021 (*n* = 2788). This is a wider age range than our symptomatic cohort, as this is a physiological dataset aiming to represent normal prostate growth across adulthood. The purpose is to provide contextual comparison to the symptomatic group and investigate the need for the existing different age‐related thresholds for normal PSA values (which are based on expected increase in volume in age). Exclusion criteria were: age (<18 and ≥90 years, *n* = 140), repeat images (*n* = 211), suspicion or confirmation of prostate pathology (from past medical history, indication that PSA level was tested, radiological evidence, biopsies, *n* = 1229), incomplete MRI coverage of prostate (*n* = 415).

### Symptomatic Patients (Cohort 2)

A separate search of the electronic database was performed for patients undergoing prostate MRI for suspected PCa for patients aged 40–79 years from October 2015 to November 2021 (*n* = 2196). Patients on this Prostate Diagnostic Cancer Pathway were symptomatic for PCa, with raised absolute serum PSA on two consecutive tests 4–6 weeks apart, with or without LUTS or an abnormal DRE. Raised PSA was defined as >3.0 ng/mL for patients aged 50–69 years (with clinical discretion for patients outside of this range), but <30 ng/mL, as beyond this level patients were at higher risk of metastatic disease, at which point staging investigations would have been more appropriate than MRI. Exclusion criteria were: patients with a previous history of PCa (seven patients), repeat imaging (95); insufficient clinical data available (four); prior treatment for benign prostatic disease (including TURP, holmium laser enucleation of the prostate or water vapour thermal therapy [e.g., Rezum]; taking finasteride or dutasteride; 51); MRI Likert scores of 4 or 5 without a biopsy being performed (16); inflammatory change ≤6 months post‐MRI (either clinically diagnosed or based on biopsy; 185); age (<40 and >79 years, three). Total PSA levels were recorded based on the sample taken within 3 months of MRI and, in cases of multiple readings, the one closest to the time of imaging. All patients underwent 1.5‐ or 3‐T scanning (GE Healthcare, Chicago, IL, USA) following a Prostate Imaging‐Reporting and Data System (PI‐RADS) compliant protocol and including T1, multiplane T2, diffusion‐weighted imaging, and dynamic contrast‐enhanced MRI [11].

## Patients and Methods

### The PVs

The PVs derived from T2‐weighted images in two different planes were selected, and the maximum anteroposterior (a), transverse (b) and longitudinal (c) diameters were measured directly on the MRI slices and applied to the clinically approved elliptical formula: PV (mL) = 0.00052 × *a* × *b* × *c* [12]. The data were stratified by age groups conforming to those used by NICE [7] (Table S1). For both cohorts, age group‐specific outliers (*n* = 33 from Cohort 1; *n* = 83 from Cohort 2), were calculated as *x* > mean + 2 sd or *x* < mean – 2 sd.

### Data Analysis

The PVs for each group were plotted against age, and a machine‐learning pipeline (repeated k‐fold cross validation) was used to ascertain which, of several fits (polynomials orders 1–10 and a spline regression) would best describe the relationship between PV and age in these data and unseen data, for each group. Outlier exclusion was performed separately for prostate volume, PSA, and PSA‐density using analysis‐specific criteria appropriate to each variable, and applied prior to statistical modelling. From this, models were made that best described PV cross‐sectionally over the age ranges of each cohort that would also take into account the data of future patients (Methods in Appendix S1). If repeated k‐fold cross validation suggested a model that was non‐linear, the *R* ^2^ of that regression was tested for statistical significance over a linear model with paired *t*‐tests, nested model *f*‐tests and the effect size calculated with Cohen's *d*. This approach was taken to avoid the classical overfitting that accompanies classical descriptive statistics. For the asymptomatic disease‐free group, this age range was the entire adult lifetime, to inform our understanding of baseline prostate physiology. To determine how PV changes with different presentations and pathology, we then use this same machine‐learning analysis to describe the PV changes in our diseased groups.

In the symptomatic groups, we analysed PSA with linear regression to determine how it is affected by both age, and disease severity. We then determined how PSA‐D was influenced by the same variables, again with linear regression and compared our findings to the current NICE thresholds. For the symptomatic groups, the data were narrowed down to the age ranges that are currently most relevant as periods of disease occurrence. Logistic regression analysis was done to test the ability of PV, PSA and PSA‐D for predicting csPCa. Youden's index was calculated to determine the optimal PSA‐D threshold.

All the statistical and graphical analyses were performed using the coding languages ‘R’ (version 4.5.1; R Foundation for Statistical Computing, Vienna, Austria) and GraphPad Prism (version 9.3.1; GraphPad Software Inc., San Diego, CA, USA). The symptomatic cohort (Cohort 2) was grouped based on their MRI and biopsy results: csPCa if Gleason Score ≥7 on biopsy; csPCa (Gleason Score 3 + 3), or benign: if negative MRI, i.e., Likert score of 1 or 2 without biopsy, or Gleason Score 6 on biopsy; or clinically benign. Clinically benign was defined as any patient who did not have confirmed PCa at the time of the investigation. This means they had a biopsy result reported as histologically benign, or atypical small acinar proliferation or prostatic intraepithelial neoplasia without a Gleason scale cancer. A biopsy was offered to patients with a Likert score or 1–2 and PSA‐D >0.15 ng/mL^2^, Likert score of 3 and PSA‐D >0.12 ng/mL^2^ or a score of 4–5 [13]. Patients with a Likert score 1–3 MRI and no biopsy were followed up for a minimum of 6 months and had at least one subsequent PSA reading [14]. Overall, the groups were as follows: asymptomatic disease free, symptomatic benign, symptomatic low‐grade PCa, symptomatic csPCa (medium‐ to high‐grade PCa). Patient numbers varied slightly between analyses due to variable data availability and analysis‐specific outlier exclusion. Each analysis included all patients with valid measurements for the relevant variable(s). Comparisons between the asymptomatic and symptomatic groups were made (between age groups 40–79 years, to avoid mixing age effects with disease effects) using the Kruskal–Wallis test with the Dunn‐Bonferroni *post hoc* test, to ascertain which groups were significantly different from each other.

## Results

### Demographics

Cohorts 1 and 2 had final populations of 760 and 1752, for PV analysis, after initial eligibility and exclusion criteria were applied.

In our asymptomatic cohort for PV analysis (*n* = 760), the cohort had a mean (range) age of 52.9 (18–89) years and median of 53 years.

In our symptomatic cohort for PV analysis (*n* = 1752), the cohort had a mean (range) age of: 64.5 (42–79) years and a median of 65 years. The mean (range) PSA level was of 7.2 (0.67–23.4) ng/mL and the median was 6.2 ng/mL. In all, 992 biopsies (56.6%) were benign, 760 (43.4%) had Gleason Score ≥3 + 3 disease, while 585 (77% of total cancers) harboured csPCa (Gleason Score ≥3 + 4).

### The PSA Level

**Fig. 1:**
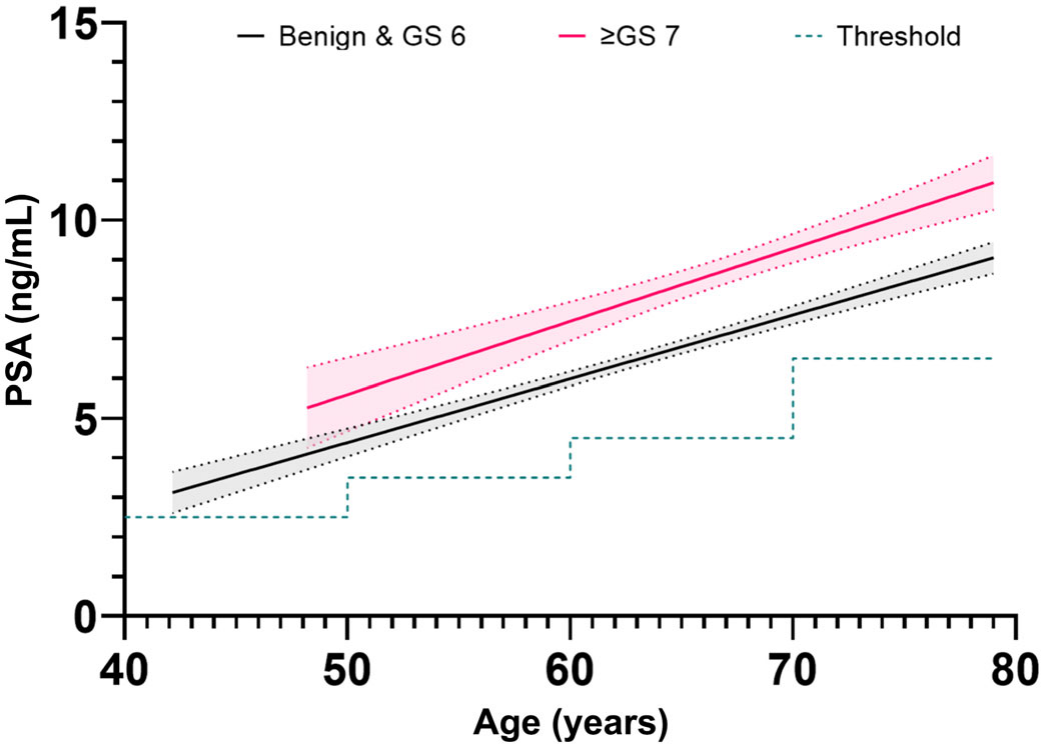
The PSA change with age in the cohort. Best fit lines represent absence (benign and Gleason Score 6) or presence (Gleason Score ≥7) of csPCa. Threshold plotted shows NICE thresholds for suspecting PCa. Shaded areas represent 95% CIs. GS, Gleason Score. The number of patients included reflects those with valid measurements for the respective analysis after application of analysis‐specific outlier filtering.

Significant positive linear relationships were shown between PSA and age in patients both with and without csPCa (*r* ^2^ = 0.09 vs 0.13, *P* < 0.001 respectively), with patients with csPCa having higher PSA levels (Fig. 1, Table S2). There was an overlap of 95% CIs below the age of ~51 years, and so the difference between PSA below this age may lose statistical significance. Between patients with and without csPCa, the rate of change in PSA with age was not significantly different (*P* = 0.34), and hence a combined gradient could be calculated, showing a gradational PSA increase of 0.17 ng/mL/year. Analysis of PSA and odds of PCa by logistic regression, demonstrated that as PSA increases by 1 ng/mL the odds of PCa increase by a factor of 1.18 (95% CI 1.14–1.21). PSA analysis included all symptomatic patients with available PSA measurements following PSA‐specific outlier exclusion.

### The PV

#### Asymptomatic, Disease‐Free Prostates (Cohort 1)

**Fig. 2:**
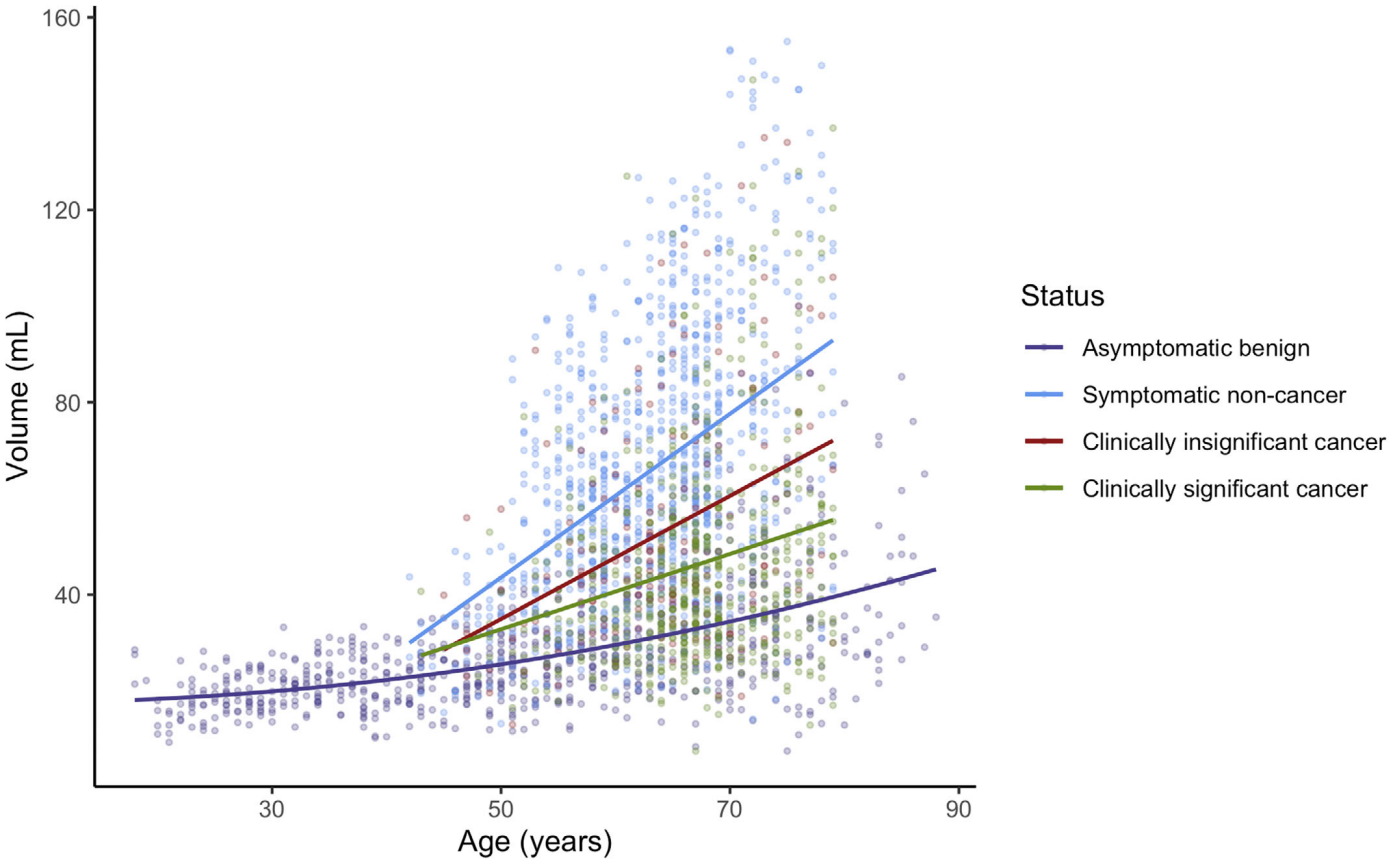
The PVs as a function of age. The optimal regression models overlaid are defined by individual repeated k‐fold cross validations for each clinical group. The number of patients included reflects those with valid measurements for the respective analysis after application of analysis‐specific outlier filtering.

The median PV of the cohort was 25.4 mL. The mean PV per age‐group continuously increased over the age‐range, from 18.7 mL to 41.3 mL (Table 1 [7], Fig. S2), with a significant positive correlation between PV and age, confirmed by Spearman's rank of rho = 0.56, *P* < 0.001. Prostate growth was estimated from the substitution of age into the differentiation of the equation of our quadratic model and increased continuously from 0.10 mL/year at age 18 years to 0.68 mL/year at age 89 years (Table S3). With repeated k‐fold cross validation, these PVs are best modelled by a quadratic curve (*R* ^2^ = 0.32) (Fig. S3A). This was verified to be a statistically significant improvement on the linear model (*f*‐test *P* < 0.005), with a large effect size (Cohen's *d* = 4.3). This translates to 32% of the variation in PV being accounted for by the variation in age. Therefore, in asymptomatic men free of prostatic disease, PV changes non‐linearly over the entire adult lifetime (Fig. 2). From the spline model, we could identify that a distinct change in the increment of PV increase occurs at the age of 48.8 years (SE 5.14) (Fig. S4). Overall, however, further repeated cross‐validation revealed that the quadratic model slightly surpassed the spline in its ability to fit the data (the latter *R* ^2^ = 0.31).

**Table 1:** Age‐stratified PVs, together with the number of patients within each group, and NICE thresholds for PSA stratified by age group.

Age ranges, years	PV, mL, mean (standard error)				*N*				PSA thresholds, μg/L, as defined by NICE [7]
	Asymptomatic disease free	Symptomatic non‐cancer	Clinically insignificant PCa	Clinically significant PCa	Asymptomatic disease free	Symptomatic non‐cancer	Clinically insignificant PCa	Clinically significant PCa	
18–29	18.7 (0.47)	–	–	–	88	–	–	–	Use clinical judgement
30–39	21.6 (0.48)	–	–	–	112	–	–	–	Use clinical judgement
40–49	23.2 (0.55)	31.5 (1.56)	31.9 (4.74)	34.8 (4.40)	117	35	7	6	>2.5
50–59	27.0 (0.62)	56.7 (1.19)	44.1 (2.83)	39.4 (1.72)	150	281	37	75	>3.5
60–69	32.2 (1.06)	66.8 (1.09)	52.1 (2.15)	43.6 (1.01)	128	520	94	313	>4.5
70–79	36.4 (1.32)	87.8 (2.67)	68.0 (5.25)	53.3 (1.96)	126	156	37	191	>6.5
80–89	41.3 (2.87)	–	–	–	39	–	–	–	Use clinical judgement

#### Prostates Symptomatic for PCa (Cohort 2)

The symptomatic group had a higher median PV of 52.0 mL. Volume analysis included all symptomatic patients with valid MRI‐derived prostate volume measurements following volume‐specific outlier exclusion. As in the asymptomatic cohort, PV increased with age. Repeated k‐fold cross validation suggested that the symptomatic benign group was best modelled by a fifth order polynomial (Fig. S3B), which was a statistically significant improvement (*f*‐test *P* < 0.05) with a modest effect size (Cohen's *d* = 2.2). However, unlike the quadratic model for the asymptomatic data, a polynomial of this order is inappropriate for interpretation clinically, and the marginal gain in explained variance does not justify the substantial increase in model complexity. By the principle of parsimony, a linear model (*R* ^2^ = 0.20) would afford minimal loss in predictive performance, but improved interpretability and clinical utility. The csPCa group was also best modelled linearly (*R* ^2^ = 0.07), although PV was consistently lower and increased with age at a slower rate (Fig. S3D). The optimal model for csPCa predicted by repeated k‐fold cross validation was a quadratic (*R* ^2^ = 0.18, Fig. S3C); however, this was not a statistically significant improvement on the linear model (*f*‐test *P* = 0.299). Overall, all the symptomatic cohorts are modelled in this study with a linear regression (Fig. 2).

Aside from age, the second variable of disease severity, also influenced PV (Fig. 2). The Kruskal–Wallis rank‐sum test, after age‐matching (40–79 years) indicated that there were significant differences in PV between our four groups (chi‐squared = 805.23, *P* < 0.001). The *post hoc* pairwise comparisons using the Bonferroni correction revealed that for each pair, all possible comparisons were statistically significant (Table S4). Notably, there was still a clear distinction between the PVs in clinically insignificant PCa and csPCa (adjusted *P* < 0.005) and between clinically insignificant PCa and symptomatic non‐cancer (adjusted *P* < 0.001).

Based on logistic regression with asymptomatic men excluded, our results demonstrated that as PV increases by 1 ml in symptomatic men, the odds of having csPCa rises by a factor of 0.97 (95% CI 0.963–0.973). This would indicate that as PV increases, the odds of having csPCa decrease. For a 10 mL increase, the odds would be ~72% of the original odds (a 28% reduction).

### The PSA‐D

**Fig. 3:**
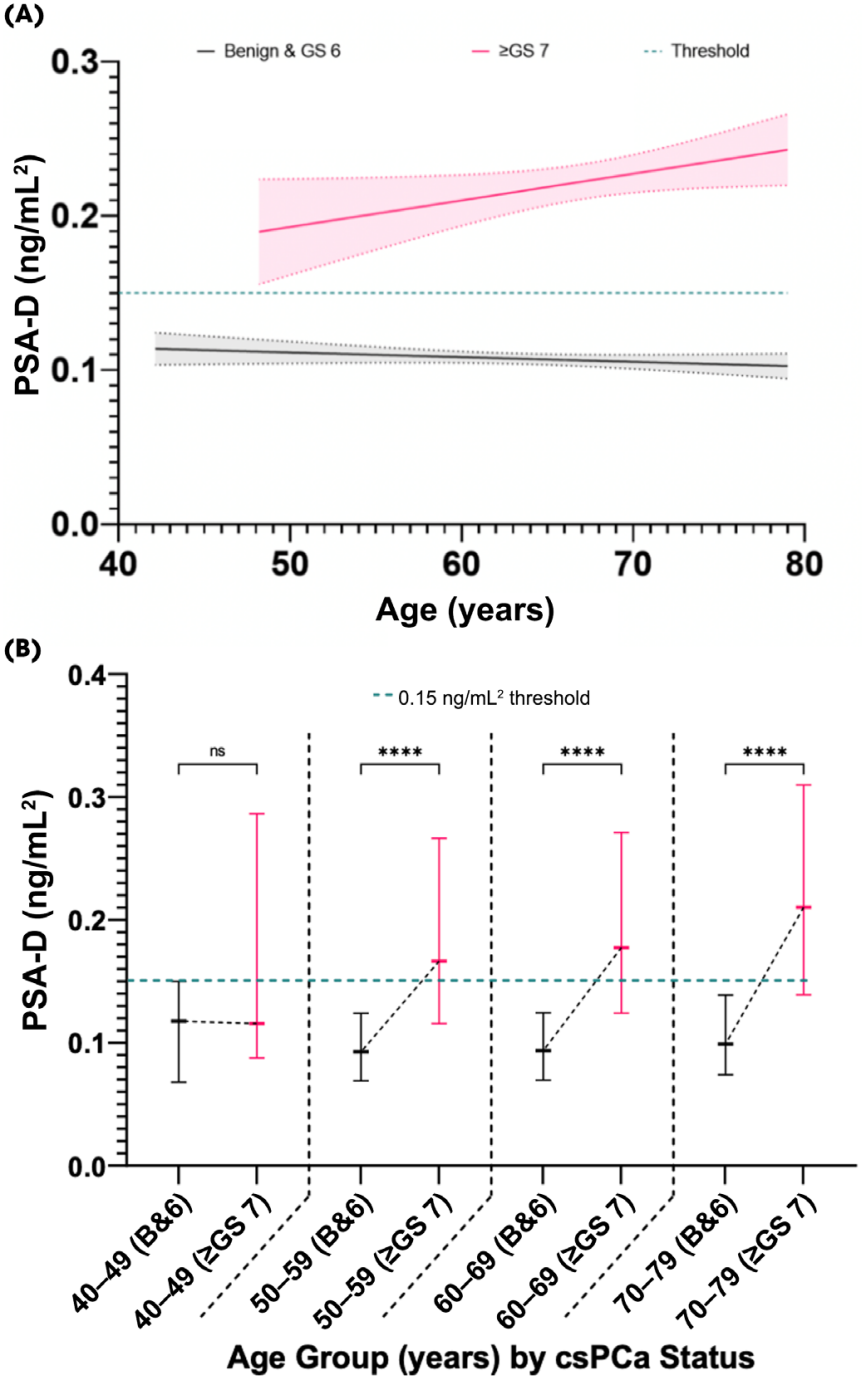
The PSA‐D change with age in the cohort. **(A)** Best fit lines represent absence (benign and Gleason Score 6) or presence (Gleason Score ≥7) of csPCa. Threshold represents the clinically recommended PSA‐D value of 0.15 ng/mL^2^. Shaded areas represent 95% CIs. **(B)** Comparisons of PSA‐D medians with interquartile ranges within age groups segregated by csPCa status. Putative 0.15 ng/mL^2^ threshold is plotted. GS, Gleason Score; ns, not significant (*P* > 0.05); ****P ≤ 0.001. The number of patients included reflects those with valid measurements for the respective analysis after application of analysis‐specific outlier filtering.

PSA‐density analysis included only patients with both PSA and prostate volume measurements available and passing respective outlier filtering criteria. Only the csPCa group, and not those with benign or low risk cancer, showed a significant linear increase in PSA‐D with age (*r* ^2^ = 0.007 vs 0.001, *P* = 0.046 vs *P* = 0.202, respectively) of 0.0017 ng/mL^2^/year. The causes of raised PSA‐D are multifactorial, but this suggests that, critically, for men with csPCa, 0.7% of this variation in PSA‐D can be explained by age (*P* = 0.046), For those without csPCa, PSA‐D remained consistently lower than csPCa at all ages (42–79 years) at around 0.114 (95% CI 0.10–0.12) ng/mL^2^ at the age of 42 years to 0.10 (95% CI 0.09–0.11) ng/mL^2^ at the age of 79 years. This allowed those with and without csPCa to be clearly distinguished using the suggested NICE clinical threshold of 0.15 ng/mL^2^ (Fig. 3), sensitivity for which was calculated as 61.7% (95% CI 57.5–65.6%), and specificity 85.4% (95% CI 83.2–87.3%). Logistic regression analysis of PSA‐D demonstrated that at thresholds of 0.10, 0.12 and 0.15 ng/mL^2^, the probability of having PCa was 0.19, 0.25 and 0.34 respectively. A PSA‐D of 0.13 ng/mL^2^ may represent a better threshold, having the greatest Youden's index with a sensitivity and specificity of 72.9% (95% CI 69.0–76.4%) and 77.9% (95% CI 75.5–80.3%), respectively. Further sensitivities and specificities at different thresholds, as used in other studies [15, 16, 17] are demonstrated in Table S5.

When grouped by decade of life, patients with csPCa in each group aged 50–79 years had significantly greater PSA‐D than patients with no PCa or Gleason Score 3 + 3 disease (*P* < 0.001 for each), reflected by a higher PSA and lower PV. However, there was no significant difference in those with and without csPCa within the 40–49 years age group (*P* = 0.14), although this may be accounted for by the relative lack of data points in this group (Fig. 3).

## Discussion

This study analysed how both age, and severity of disease affect three prostate‐related measurements: PV, PSA and PSA‐D. We demonstrated that the healthy prostate undergoes a non‐linear increase in volume with age. In the symptomatic groups, we also found PV increased with age, with significant differences when stratified by disease severity. Additionally, for symptomatic patients, PSA increased with age regardless of whether clinically significant disease was present; however, patients with csPCa had consistently higher PSA levels. PSA‐D only demonstrated a statistically significant increase for the csPCa group, and for all ages, the PSA‐Ds for this group were above the NICE recommended PSA‐D threshold.

To date, few physiological studies have mapped how the PV changes across the lifespan of asymptomatic patients, but that which exists, agrees with our asymptomatic PV results [18, 19, 20, 21, 22, 23, 24]. Our study of disease‐free patients benefited from a large sample size, analysis of all ages across the adult lifetime, and use of the highest clinical standard of MRI for more accurate measurements [25, 26, 27]. The majority of prior studies employed TRUS for PV measurements [19, 20, 21, 22, 23, 24, 27], and investigated men aged >40 years [19, 20, 22, 23, 24], and of the few that did study younger males, the sample size was comparatively small [18, 21, 28]. Notably, in the existing literature there is differing opinion as to whether PV changes are linear [18], or better modelled with high‐degree polynomials [19]. We applied a cross‐validated machine learning approach to derive our quadratic model, which, by avoiding overfitting, produced a higher quality of fit for our model of asymptomatic PV compared to the non‐validated approaches, thus our findings should be applicable to future patient cohorts.

The symptomatic, non‐cancer group had the highest mean PV, with the demonstrated increases in PV with age consistent with the existing literature [29, 30]. Patients developing BPH are more likely to present to primary care with LUTS, leading to a raised PSA test and urological referral. Conversely, in patients with csPCa, as the Gleason score increases, PV tended to decrease. This is in line with the limited prior studies in patient cohorts, with csPCa being associated with smaller gland volumes [31, 32]. However, mean PVs in men with csPCa do not decrease below those of disease‐free, asymptomatic men, thus distinction between the models remains preserved.

Given the known relationship of PSA with gland volume, the dynamic changes in PV with age across all the cohorts would be broadly supportive of setting age‐related PSA thresholds. We found PSA to increase with age, with the most marked increase between consecutive age groups being 60–69 to 70–79 years, both for those with and without csPCa. However, it is notable that the median PSA per decade in our symptomatic cohort was consistently greater than the age‐stratified thresholds recommended by NICE [7]. This may be heavily influenced by selection bias, as a threshold limit of at least 3.0 ng/mL is typically applied at the time of referral, reducing the potential number of patients with lower PSA levels in this study.

Conversely, we found that PSA‐D does not significantly increase between consecutive age groups, thus a static threshold could be employed. PSA‐D was significantly elevated in all age groups 50–79 years for patients with csPCa, consistent with previous studies [33, 34]. This reflects the significantly higher PSA and smaller PVs in these patients. Our results show that for PSA‐D, the currently employed clinical threshold of 0.15 ng/mL^2^ is supported, although the more conservative value of <0.13 ng/mL^2^ demonstrated a better statistical cut‐off for benign disease.

Our study has some limitations, including the retrospective and cross‐sectional design; ideally the same cohorts of men would be followed‐up in a longitudinal study, with multiple repeat MRIs to map the changes across individuals. We cannot fully exclude the presence of undetected PCa in the disease‐free asymptomatic cohort; however, we reviewed the medical notes and excluded patients with a prior PSA test, or urological referral. Likewise, the presence of PCa cannot be excluded in MRI‐negative patients not undergoing biopsy; however, clinical follow‐up has been employed for this purpose in other studies [29, 35]. Furthermore, any biopsy procedure is prone to sampling error and may have under‐ or over‐estimated the presence of csPCa in some patients. This was not a formal screening study, therefore patients referred to urology were highly likely to have symptoms, and/or a raised PSA above a threshold of 3.0 ng/mL, leading to higher median PSA levels than may have been expected across this cohort. MRI may under‐estimate PV compared to the ‘gold standard’ of prostatectomy; however, it is known to outperform abdominal ultrasound or TRUS or CT and represents the best clinical reference standard [25]. Our study additionally benefits from the large sample size and mix of patients across age‐groups and disease states.

## Conclusions

Our study maps the increases in PV with age across symptomatic and asymptomatic men and demonstrates how the presence and grade of disease can affect PV. We provide evidence for the use of age‐related PSA thresholds and establish that a static PSA‐D threshold can be applied across age groups to help differentiate presence of csPCa from benign, low‐grade, or the absence of PCa.

## Disclosure of Interests

Nothing is declared.

## Statement of Originality

We confirm that the material in this paper is not copied, nor a paraphrase or abstract from any published material, unless it is clearly identified as such and a full source reference given. This paper has not been published elsewhere.

## Supporting information


**Table S1.** The NICE defined thresholds for PSA [7] stratified by age group.
**Table S2.** The PSA vs age range for all groups. Median PSA (ng/mL) values listed, with interquartile range in brackets.
**Table S3.** Prostate growth rate by age in asymptomatic men.
**Table S4.** Age stratified PVs, together with number of patients within each age‐group, and results for the Dunn–Bonferroni post hoc test. *P* < 0.05 was considered to be statistically significant (*0.01 < *P* < 0.05; **0.001 < *P* < 0.01; ***0.0001 < *P* < 0.001: ****P < 0.001).
**Table S5.** Comparison of different sensitivities and specificities of PSA‐D thresholds from different studies, for determining csPCa across ages 40–79. 95% confidence intervals in brackets.
**Fig. S1.** CONSORT diagram explaining study participants and reasons for exclusions.
**Fig. S2.** Mean PV per age‐group for asymptomatic patients.
**Fig. S3.** Repeated k‐fold cross validations for each cohort group, with error bars showing standard errors (A) asymptomatic disease free (B) Symptomatic non‐cancer (C) Clinically insignificant cancer (D) Clinically significant cancer.
**Fig. S4.** Spline model of the PV data from asymptomatic patients (*R* ^2^ = 0.31). This model may indicate a point where a distinct change in the increment of PV increase occurs (48.84, SE ± 5.14 years).
