# Supplementary material for: The influence of age and the presence of prostate cancer on prostate volume, PSA and PSA density
Source: BJU Int. 2026 Feb 23;137(5):805–12. doi: 10.1111/bju.70169 (PMC13071546; doi:10.1111/bju.70169)
Supplement: Supplementary file 1 — Table S1. The NICE defined thresholds for PSA [7] stratified by age group. Table S2. The PSA vs age range for all groups. Median PSA (ng/mL) values listed, with interquartile range in brackets. Table S3. Prostate growth rate by age in asymptomatic men. Table S4. Age stratified PVs, together with number of patients within each age‐group, and results for the Dunn–Bonferroni post hoc test. P < 0.05 was considered to be statistically significant (*0.01 < P < 0.05; **0.001 < P < 0.01; ***0.0001 < P < 0.001: ****P < 0.001). Table S5. Comparison of different sensitivities and specificities of PSA‐D thresholds from different studies, for determining csPCa across ages 40–79. 95% confidence intervals in brackets. Fig. S1. CONSORT diagram explaining study participants and reasons for exclusions. Fig. S2. Mean PV per age‐group for asymptomatic patients. Fig. S3. Repeated k‐fold cross validations for each cohort group, with error bars showing standard errors (A) asymptomatic disease free (B) Symptomatic non‐cancer (C) Clinically insignificant cancer (D) Clinically significant cancer. Fig. S4. Spline model of the PV data from asymptomatic patients (R 2 = 0.31). This model may indicate a point where a distinct change in the increment of PV increase occurs (48.84, SE ± 5.14 years). [file BJU-137-805-s001.docx]

**Supplemental data**

**Methods**

**The machine learning approach**

Classical descriptive statistics enable only interpolation within the given study cohort. Regressions to describe the data will fit the data better as the degree of the polynomial increases, however this commonly leads to overfitting. By contrast, with our machine learning approach, we train the models on our data having taken out a sample, which we then test our model against iteratively, in a process called repeated k-fold cross validation. This means our model best describes the relationship in our own cohort, but also the relationship for unseen, future patients. The optimal model would therefore not necessarily be a higher degree polynomial. If repeated k-fold cross validation suggested a model that was non-linear, the R2 of that regression was tested for statistical significance over a linear model with paired t-tests, nested model f-tests and the effect size calculated with Cohen’s d.

**Supplemental Tables**

| **Age ranges (years)** | **NICE defined PSA thresholds (ng/mL)** |
| --- | --- |
| **<40** | Use Clinical judgement |
| **40-49** | > 2.5 |
| **50-59** | > 3.5 |
| **60-69** | > 4.5 |
| **70-79** | > 6.5 |
| **80-89** | Use Clinical judgement |

**Table S1.** NICE defined thresholds for PSA(7) stratified by age-group.

|  | **Age** | | | |
| --- | --- | --- | --- | --- |
|  | **40-49** | **50-59** | **60-69** | **70-79** |
| NICE threshold | 2.5 | 3.5 | 4.5 | 6.5 |
| All patients [n=1747] | 3.44 (1.68) | 4.78 (3.03) | 5.92 (3.29) | 8.42 (4.84) |
| Benign [n=1019] | 3.36 (1.24) | 4.63 (2.66) | 5.60 (2.83) | 8.3 (4.83) |
| Benign & GS 6 [n=1193] | 3.36 (1.55) | 4.65 (2.68) | 5.61 (2.83) | 8.09 (4.19) |
| ≥GS 6 (Any PCa) [n=728] | 4.37 (1.80) | 5.53 (3.92) | 6.55 (4.2) | 8.56 (4.91) |
| ≥GS 7 (csPCa) [n=554] | 5 (2) | 5.79 (4.04) | 6.9 (4.71) | 9.00 (5.48) |

**Table S2.** PSA versus Age range for all groups. Median PSA (ng/ml) values listed, with interquartile range in brackets.

| **X = Age (years)** | **Growth rate  (Y = 0.0082x – 0.05) (ml/year)** |
| --- | --- |
| 18 | 0.10 |
| 29 | 0.19 |
| 39 | 0.27 |
| 49 | 0.35 |
| 59 | 0.48 |
| 69 | 0.52 |
| 79 | 0.60 |
| 89 | 0.68 |

**Table S3.** Prostate growth rate by age in asymptomatic men.

| **Age ranges (yrs.)** | **Mean prostate volume (ml) +- standard error** | | | | **N=** | | | |
| --- | --- | --- | --- | --- | --- | --- | --- | --- |
|  | Asymptomatic disease free | Symptomatic non-cancer | Clinically Insignificant cancer | Clinically Significant cancer | Asymptomatic disease free | Symptomatic non-cancer | Clinically Insignificant cancer | Clinically Significant cancer |
| **18-29** | 18.7 (+- 0.47) | - | - | - | 88 | - | - | - |
| **30-39** | 21.6 (+- 0.48) | - | - | - | 112 | - | - | - |
| **40-49** | 23.2 (+- 0.55) | 31.5 (+-1.56) | 31.9 (+-4.74) | 34.8 (+-4.40) | 117 | 35 | 7 | 6 |
| **50-59** | 27.0 (+- 0.62) | 56.7 (+-1.19) | 44.1 (+-2.83) | 39.4 (+-1.72) | 150 | 281 | 37 | 75 |
| **60-69** | 32.2 (+- 1.06) | 66.8 (+- 1.09) | 52.1 (+-2.15) | 43.6 (+-1.01) | 128 | 520 | 94 | 313 |
| **70-79** | 36.4 (+- 1.32) | 87.8 (+- 2.67) | 68.0 (+-5.25) | 53.3 (+-1.96) | 126 | 156 | 37 | 191 |
| **80-89** | 41.3 (+-2.87) | - | - | - | 39 | - | - | - |
| **Dunn Bonferroni pairwise comparisons for ages 40-79 (Adjusted p-values)** | p = 3.18e^-172^ (=0.0000)  p =1.64e^-33^ (=0.0000) |  | p = 4.47e^-3^ (=0.0045)  p =9.29e^-8^ (=0.0000)  p =1.21e^-38^ (=0.0000) | p =8.5e^-44^ (=0.0000) |  |  |  |  |

**Table S4** Age stratified prostate volumes, together with number of patients within each age-group, and results for the Dunn–Bonferroni post hoc test. p < 0.05 was considered to be statistically significant (* 0.01 < p < 0.05; ** 0.001 < p < 0.01; *** 0.0001 < p < 0.001: **** p < 0.001 ).

| **PSA-D threshold (ng/ml2)** | **Reference** | **Sensitivity (%)** | **Specificity (%)** |
| --- | --- | --- | --- |
| 0.070 | Nordström et al. (17) | 96.4 (94.5 – 97.7) | 24.9 (22.4 – 27.4) |
| 0.085 | Ha et al. (16) | 91.5 (88.9 - 93.6) | 41.6 (38.9 – 44.5) |
| 0.100 | Jue et al. 2017 (15) | 87.7 (84.7 - 90.2) | 54.6 (51.7 – 57.4) |
| 0.120 | This study | 78.7 (75.1 - 81.9) | 71.2 (68.5 – 73.8) |
| 0.132 | This study | 72.9 (69.0 – 76.4) | 77.9 (75.5 – 80.3) |
| 0.150 | Jue et al. (15), Nordström et al. (17) | 61.7 (57.5 – 65.6) | 85.4 (83.2 – 87.3) |

**Table S5** Comparison of different sensitivities and specificities of PSA-D thresholds from different studies, for determining csPCa across ages 40-79. 95% confidence intervals in brackets. PSA-D = PSA-density, csPCa = clinically significant prostate cancer**.**

**Supplemental figures**

**
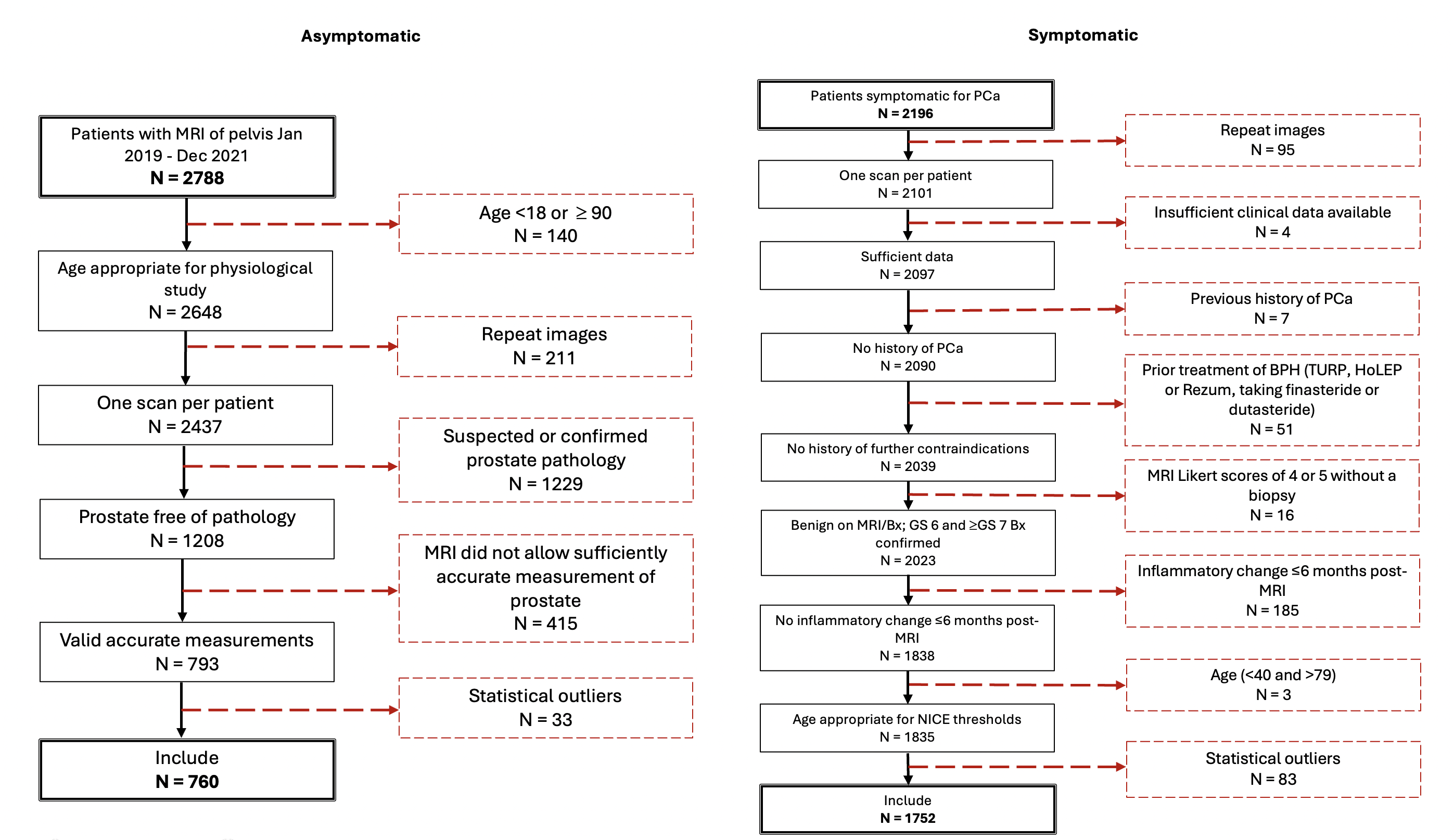
**

**Figure S1. CONSORT diagram explaining study participants and reasons for exclusions.**


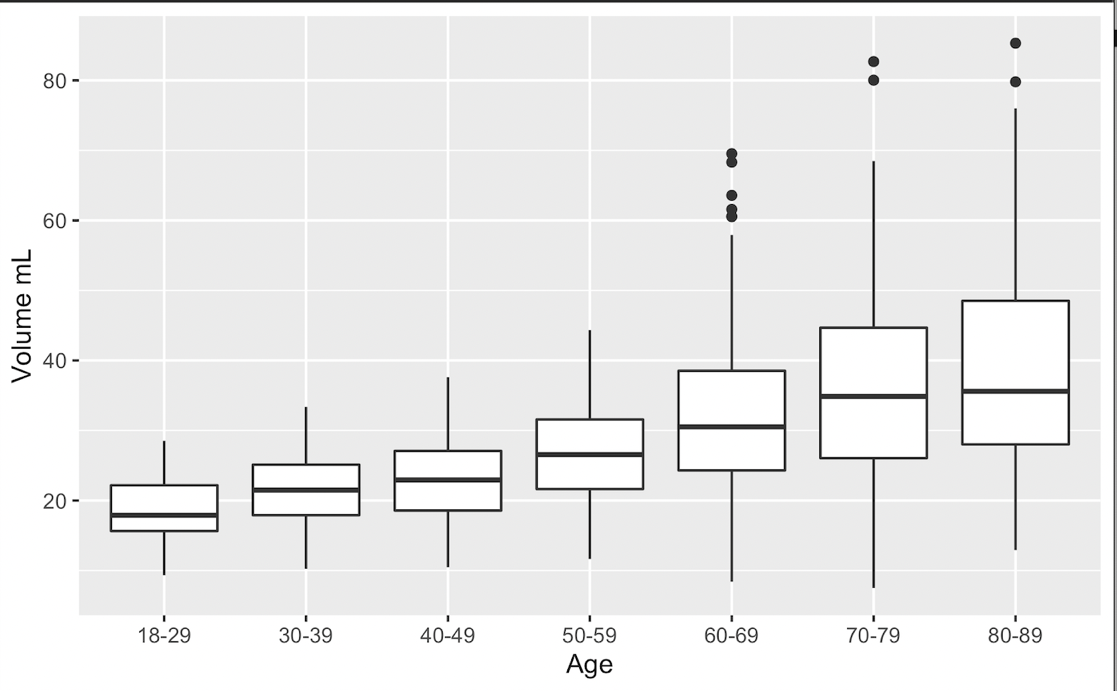


**Figure S2**. Mean prostate volume per age-group for asymptomatic patients.


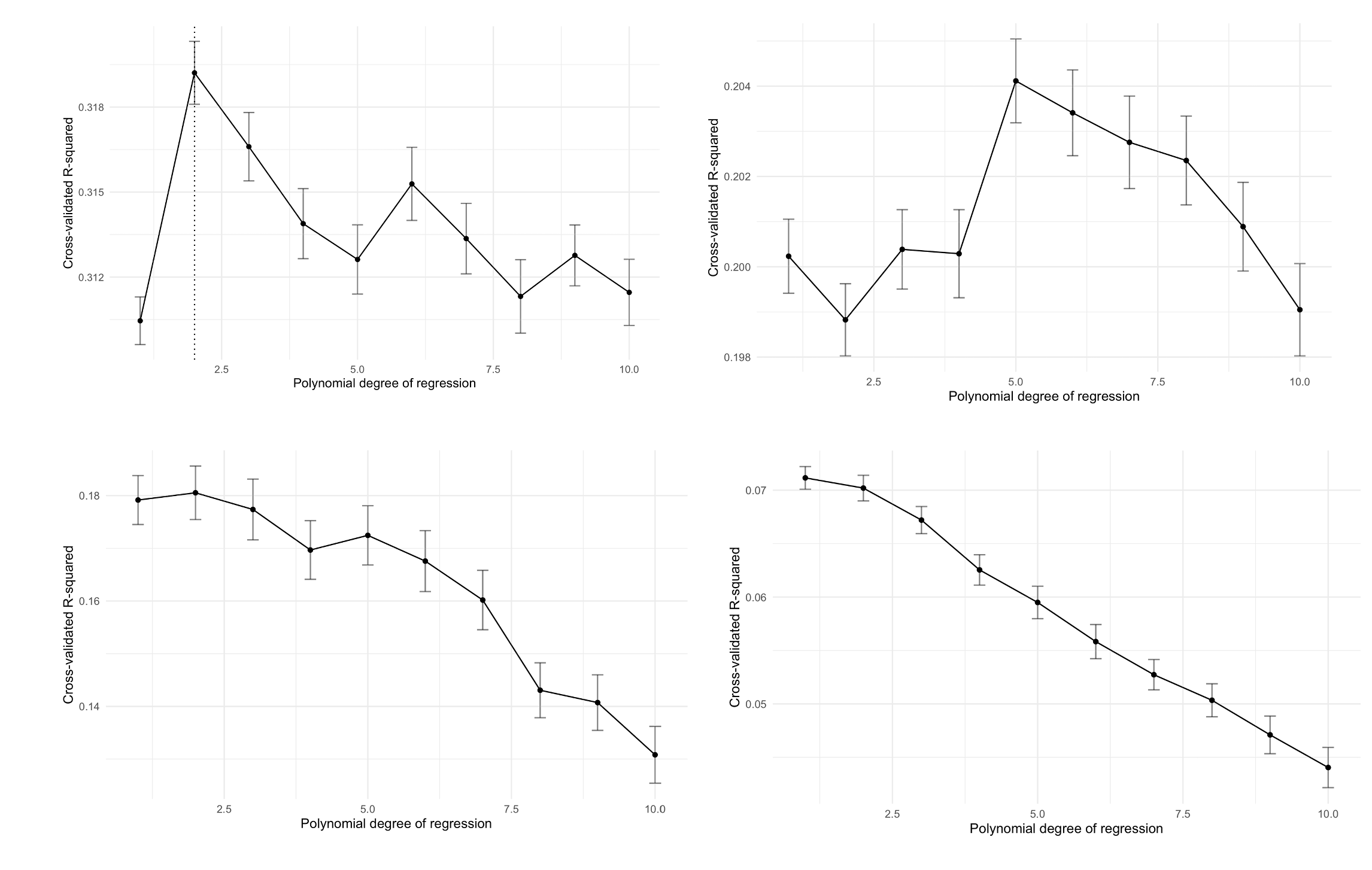


A

B

C

D

**Figure S3** - Repeated k-fold cross validations for each cohort group (A) asymptomatic disease free (B) Symptomatic non-cancer(C) Clinically insignificant cancer (D) Clinically significant cancer


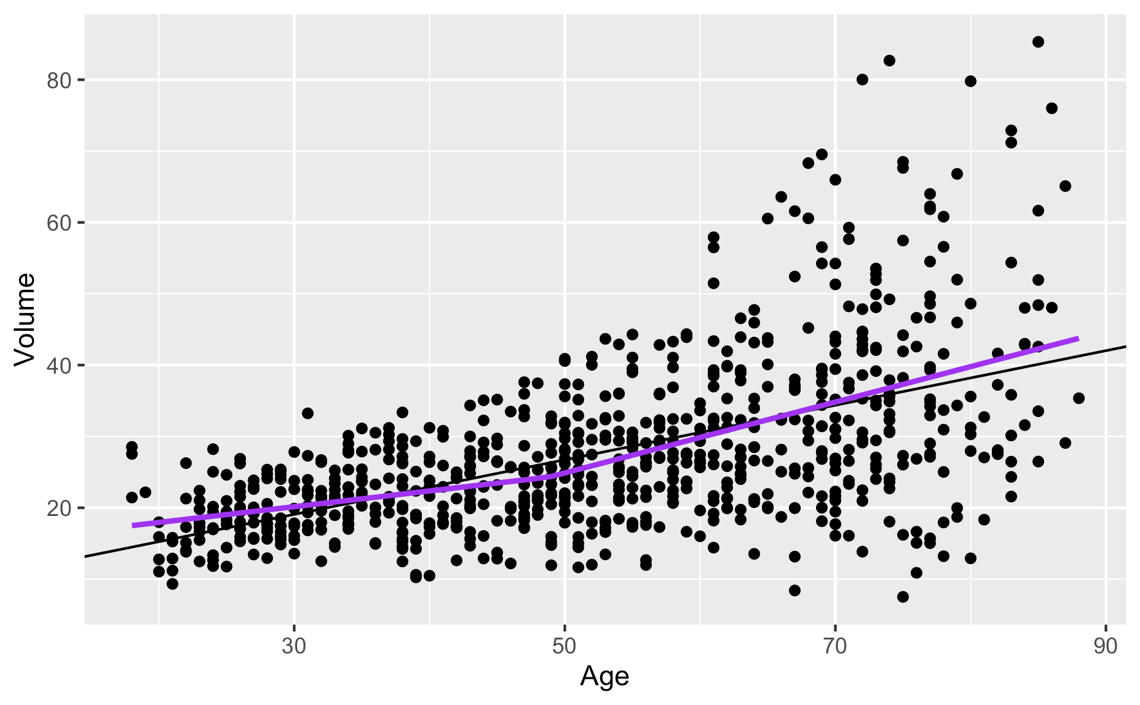


**Figure S4**. Spline model of the prostate volume data from asymptomatic patients (R^2^ = 0.31). This model may indicate a point where a distinct change in the increment of PV increase occurs (48.84 +/- 5.14 years).
